# A single genomic region involving a putative chromosome rearrangement in flat oyster (*Ostrea edulis*) is associated with differential host resilience to the parasite *Bonamia ostreae*

**DOI:** 10.1111/eva.13446

**Published:** 2022-07-21

**Authors:** Inés Martínez Sambade, Adrian Casanova, Andrés Blanco, Manu K. Gundappa, Tim P. Bean, Daniel J. Macqueen, Ross D. Houston, Antonio Villalba, Manuel Vera, Pauline Kamermans, Paulino Martínez

**Affiliations:** ^1^ Department of Zoology, Genetics and Physical Anthropology, ACUIGEN Group, Faculty of Veterinary Universidade de Santiago de Compostela Lugo Spain; ^2^ The Roslin Institute and Royal (Dick) School of Veterinary Studies Midlothian UK; ^3^ Centro de Investigacións Mariñas (CIMA) Vilanova de Arousa Spain; ^4^ Departamento de Ciencias de la Vida Universidad de Alcalá Alcalá de Henares Spain; ^5^ Research Centre for Experimental Marine Biology and Biotechnology (PIE) University of the Basque Country (UPV/EHU) Plentzia Spain; ^6^ Wageningen Marine Research Yerseke The Netherlands; ^7^ Marine Animal Ecology Group Wageningen University Wageningen The Netherlands

**Keywords:** bonamiosis, chromosome rearrangement, disease resilience, *Ostrea edulis*, SNP‐chip

## Abstract

European flat oyster (*Ostrea edulis*) is an ecologically and economically important marine bivalve, that has been severely affected by the intracellular parasite *Bonamia ostreae*. In this study, a flat oyster SNP array (~14,000 SNPs) was used to validate previously reported outlier loci for divergent selection associated with *B. ostreae* exposure in the Northeast Atlantic Area. A total of 134 wild and hatchery individuals from the North Sea, collected in naïve (NV) and long‐term affected (LTA) areas, were analysed. Genetic diversity and differentiation were related to the sampling origin (wild vs. hatchery) when using neutral markers, and to bonamiosis status (NV vs. LTA) when using outlier loci for divergent selection. Two genetic clusters appeared intermingled in all sampling locations when using outlier loci, and their frequency was associated with their bonamiosis status. When both clusters were compared, outlier data sets showed high genetic divergence (*F*_ST_ > 0.25) unlike neutral loci (*F*_ST_ not ≠ 0). Moreover, the cluster associated with LTA samples showed much higher genetic diversity and significant heterozygote excess with outlier loci, but not with neutral data. Most outliers mapped on chromosome 8 (OE‐C8) of the flat oyster genome, supporting a main genomic region underlying resilience to bonamiosis. Furthermore, differentially expressed genes previously reported between NV and LTA strains showed higher mapping density on OE‐C8. A range of relevant immune functions were specifically enriched among genes annotated on OE‐C8, providing hypotheses for resilience mechanisms to an intracellular parasite. The results suggest that marker‐assisted selection could be applied to breed resilient strains of *O. edulis* to bonamiosis, if lower parasite load and/or higher viability of the LTA genetic cluster following *B*. *ostreae* infection is demonstrated.

## 1 INTRODUCTION

The European flat oyster (*Ostrea edulis* L.) is a marine bivalve mollusc distributed from the Atlantic coast of Morocco to the Norwegian Sea and through the Mediterranean Sea up to the Black Sea (Perry et al., 2017). Due to their economic value, flat oyster populations exist outside their natural range in many different countries following human‐mediated transfer (Colsoul et al., 2021). *Ostrea edulis* is a sessile filter‐planktotrophic species (Ezgeta‐Balić et al., 2020; Peharda et al., 2012) that can reach 20 cm and live up to 20 years, preferably in firm benthic habitats comprised of mud, rocks, hard silt or even artificial substrates created with broken shells (cultch), appearing at depths up to 80 m. The flat oyster is a protandrous hermaphrodite, maturing from 8 to 10 months, depending on environmental factors. Reproduction takes place within the females' pallial cavity, where ova are fertilized by sperm that pass through the gills as part of the normal feeding process. After an incubation period between eight and 10 days, free‐swimming larvae are dispersed into the environment before metamorphosis and settlement (Bayne, 2017). Dispersal can last 10–30 days and involve distances longer than 10 km under favourable conditions (Berghahn & Ruth, 2005). Each generation tends to settle on top of the preceding one, such that reefs grow vertically and provide a habitat for other organisms (Chambers et al., 2017). This property is why oysters are one of a number of bivalve species that are considered ‘ecosystem engineers’ (Smaal et al., 2018).

Flat oyster has been considered a biomonitor to detect the impact of different contaminants in the wild (Medaković et al., 2006; Valbonesi et al., 2003) and further used as a biofilter in aquaculture for removal of dissolved and particulate nutrients from fishpond effluents (Cranford et al., 2011; Shpigel, 2005). Moreover, it is a highly appreciated gastronomic and nutritional resource, representing one of the most important aquaculture species of the 20th century. However, in the past 40 years, the production of European flat oyster has declined drastically worldwide from the peak production of nearly 30,000 t in 1961 to just 2000 t in 2012 (FAO, 2020), due to a combination of overexploitation, habitat destruction, environmental change and diseases. European flat oyster aquaculture has been increasingly replaced by other oyster species such as *Crassostrea gigas* (Troost et al., 2008) and flat oyster now accounts for <0.2% of the total global production of all farmed oyster species (5 Mt in 2015; FAO, 2020).

Various diseases impact both wild and cultured populations of *O. edulis* (Sas et al., 2020). The two main disease‐causing organisms—*Martelia refringens* (marteliosis) and *Bonamia ostreae* (bonamiosis)—started to spread in the 1970s and 1980s, respectively, and despite changes in management practices and intensive restocking programs, production has remained steady and low in recent years. *Bonamia ostreae* is a destructive intracellular parasite transmitted horizontally and putatively also vertically, causing a heavy inflammatory reaction that leads to high mortality following the loss of normal architecture and function in multiple organs (da Silva et al., 2008). The parasite proliferates inside the haemocytes occurring mostly in the connective tissue but also in epithelia of numerous organs. Due to its small size (3–5 μm in diameter) and its intracellular location, *B. ostreae* infection is often difficult to detect visually, which is why standard diagnostic methods use cytology and histopathology together with qPCR to screen oyster tissues (Sas et al., 2020). Although adult individuals appear to be the most susceptible to mortality in the field, the parasite can also infect larvae, seeds and juvenile oysters, suggesting that larvae displacement with ocean currents may result in the movement of the parasite through Europe (Arzul et al., 2011). However, the patchy distribution of the parasite in the Northeast Atlantic Ocean suggests that infection in one area does not necessarily ensure the transference of the parasite to neighbouring areas.

Understanding the genetic basis of host response to parasites and host–parasite interactions during infection is essential to ascertain the best strategy to control diseases. Several studies have supported that both in vitro contact of *B*. *ostreae* with haemocytes and its inoculation into healthy oysters activates the immune response of the host, inducing up‐regulation of immune‐related genes and modification of cellular parameters (Pardo et al., 2016; Ronza et al., 2018). Following an oligo‐microarray approach, genes involved in resistance such as antimicrobial peptides and extracellular matrix components were identified by comparing gene expression profiles of naïve and long‐term affected oyster strains (NV and LTA, respectively; Ronza et al., 2018). Breeding programs to produce oysters resistant to bonamiosis rely on the underlying genetic variation and have been successfully applied by selecting broodstock among survivors after long exposure in heavily affected areas (Ronza et al., 2018). Quantitative trait loci (QTL) and markers associated with resistance to bonamiosis have been reported, and this information could accelerate the selection process (Lallias et al., 2009). Combining functional and marker‐association genomic strategies, Harrang et al. (2015) also identified SNP (single nucleotide polymorphism)‐associated markers linked to differential expression of candidate genes (eQTL).

Recently, a SNP array containing a total of 14,065 putative flat oyster SNPs (Gutiérrez et al., 2017) and 37 SNP‐associated markers from candidate genes identified by Ronza et al. (2018) were analysed to identify signals of divergent selection to bonamiosis by comparing two sets of NV and LTA flat oyster populations (Vera et al., 2019). Within a low differentiation genomic background in the Atlantic area (*F*_ST_ = 0.0079; Vera et al., 2016), a set of 22 consistent and 87 suggestive outliers were associated with divergent selection to bonamiosis. These markers discriminated NV vs. LTA populations, providing useful information for their putative application in breeding programs to obtain parasite resistant strains. Interestingly, the strong linkage disequilibrium observed between most of these markers suggested that a major QTL could underlie resilience to the parasite.

In this study, we used the flat oyster SNP array to validate the genetic markers associated with resistance to *B. ostreae* reported by Vera et al. (2019) in a narrower sampling scenario, where populations of have been subjected to different bonamiosis pressures. We used a new chromosome‐level flat oyster genome assembly to map validated outliers and differentially expressed genes (DEG) previously reported by Ronza et al. (2018) for an integrative analysis. The results confirmed the association of outliers with the bonamiosis status of samples in an independent scenario and demonstrated their location in a single genomic region containing genes enriched in relevant immune functions over the genomic background.

## 2 MATERIALS AND METHODS

### 2.1 Sampling

**FIGURE 1:**
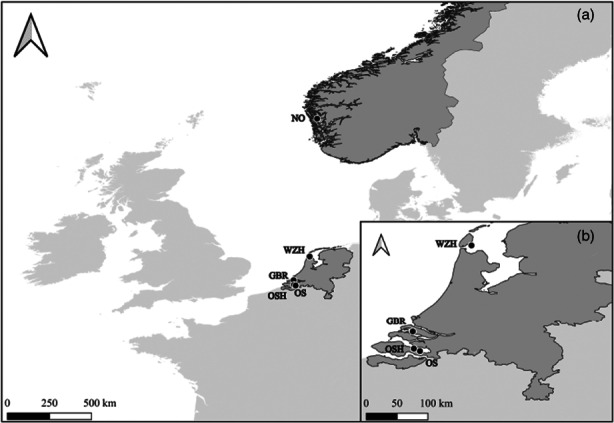
Geographical location of the *Ostrea edulis* samples: (a) all samples; (b) zoom on the Netherlands sampling area

The analysis was performed on 134 flat oyster individuals collected in 2019 from locations with different bonamiosis prevalence: (i) three oyster beds from the North Sea (OS, GBR, NO); (ii) two hatchery batches (OSH, WZH) from the same area (FAO subarea 27.4 https://www.fao.org/fishery/en/area/27/en; Figure 1) (Table 1). Hatchery spat was produced from wild individuals of the same area that were conditioned in the hatchery until spawning. Hatchery samples were included in the study considering their interest for restocking bonamiosis‐affected areas using *Bonamia*‐resilient strains from marker‐assisted selection programs. These samples provided additional information for the association analysis and validation of markers, and specifically in one case, the Wadden Sea, the only representative sample was from the hatchery. Furthermore, hatchery data allowed an estimation of the contribution of parents for checking the reproduction protocols in batches at hatcheries to maintain genetic diversity in future restoration programs. The sampling locations were sorted according to bonamiosis status from previous information as: (i) naïve (NV), supposedly to have never been in contact with the parasite and (ii) long‐term affected (LTA), where parasite presence was first reported over 30 years ago and monitored over time to the present (Engelsma et al., 2010).

**TABLE 1:** Characteristics of *Ostrea edulis* samples analysed in this study

Sample code	Location	Country	Origin	*N*	Bonamiosis status
OS	Oosterschelde	The Netherlands	Wild	28	LTA
OSH	Oosterschelde	The Netherlands	Hatchery	28	LTA
GBR	Grevelingen	The Netherlands	Wild	21	LTA
WZH	Waddenze	The Netherlands	Hatchery	27	NV
NO	Bergen	Norway	Wild	30	NV

Abbreviations: LTA, long‐term affected areas; NV, Naïve areas.

### 2.2 DNA extraction and SNP genotyping

Genomic DNA was extracted from gill tissue using the E.Z.N.A.® Mollusc DNA kit (OMEGA Bio‐Tek) following the manufacturer's recommendations. Briefly, between 30 and 50 mg of fresh gill was treated with a solution containing 20 μl of proteinase K and 700 μl of lysis buffer (CSPL) overnight while gently shaking to mix thoroughly at 65°C. Samples were subjected to RNAse A treatment and to several buffer washes by centrifugation. DNA quantity and purity was measured with a Nanodrop spectrophotometer and DNA concentrations were normalized for genotyping.

Samples were shipped to Identigen (Dublin, Ireland) for genotyping on a medium‐density Affymetrix Axiom SNP array (14,950 SNPs; 1 SNP per ~63 kb of the flat oyster genome; 935.6 Mb; see below). After filtering SNPs with <90% call rate, 11,641 were retained, which supports the robustness of the SNP chip platform (Gutiérrez et al., 2017). A total of 611 SNPs were monomorphic across all sampling locations and discarded for structure analyses, as they would not influence the results. Three different SNP data sets were considered according to the analyses performed (Section 3) and the information previously reported by Vera et al. (2019): (i) the whole data set, (ii) the two outlier data sets related to bonamiosis resistance according to more or less stringent statistical criteria (consistent and suggestive, respectively), and (iii) the neutral data set.

### 2.3 Genetic diversity

Genetic diversity per sample was estimated using expected (*H*_e_) and observed (*H*_o_) heterozygosity, and allelic richness (Ar), computed using the rarefaction method. These analyses were performed with the DiveRsity R package v. 1.9 using the ‘basicStats’ function (Keenan et al., 2013). Non‐parametric Mann–Whitney and Kruskal–Wallis tests were performed to check for genetic diversity differences between pairs or groups of samples, respectively.

Conformance to Hardy–Weinberg expectations was evaluated with an exact test implemented in the R package Genepop v 1.1.7 (Rousset, 2008). This test was performed using the complete enumeration method to estimate *p*‐values (Louis & Dempster, 1987). Global *p*‐values per sample were obtained with Fisher's method (Fisher, 1933). Deviations from panmixia were estimated for each locus and averaged across loci for each sampling location using the intrapopulation fixation index (*F*_IS_; Wright, 1949). The significance of *F*_IS_ was tested with the 95% bias corrected confidence intervals (CI) obtained after 1000 bootstraps iterations with ‘divBasic’ function (DiveRsity R package). A full‐pedigree reconstruction method implemented in Colony v2.0.6.6 (Wang, 2004) was applied to estimate parentage, and the number of breeders in all locations/hatchery batches. We used 1000 highly polymorphic SNPs (Minimum Allele Frequency [MAF] > 0.35), homogeneously distributed across the genome, among the 11,641 SNPs available due to the computational limits of the program working with thousands of markers. The maximum likelihood method implemented in Colony was also used to estimate effective population size assuming random mating.

### 2.4 Genetic structure

Pairwise interpopulation fixation index (*F*_ST_) between sampling locations was calculated using the StaMPP R package v 1.6.2 with the ‘stamppFst’ function (Pembleton et al., 2013); 10,000 bootstrap replicates across loci were used to generate 95% confidence intervals and *p*‐values regarding the null hypothesis (*F*_ST_ = 0). Global *F*_ST_ was calculated using the R package Genepop with the ‘Fst’ function (Raymond & Rousset, 1995).

The number of different genetic clusters in our sampling collection was evaluated with STRUCTURE v 2.3.4 (Pritchard et al., 2000). This program looks for the number of different genetic clusters (*K* value) in the samples compatible with the data by analysing linkage disequilibrium and Hardy–Weinberg equilibrium following a Bayesian clustering approach. Tests without a priori information were performed using the R package ParallelStructure v 1.0 (Besnier & Glover, 2013) with a burn‐in of 100,000 iterations and 200,000 Markov Chain Monte‐Carlo steps. Ten independent replicate runs were used to increase precision. The ADMIXTURE ancestry model was performed with K1 − K6 (*N* samples +1). To identify the most likely number of clusters, two different K estimators were used: deltaK (Evanno et al., 2005) and Mean LnP(K) (Pritchard et al., 2000). StructureSelector Web‐based software (Li & Liu, 2018) was used to obtain K estimators and CLUMPAK graphical outputs (Kopelman et al., 2015). Moreover, an analysis with a priori population information was performed (POPINFO = 1) on our samples with *K* = 2 using consistent and suggestive outliers taking as references the LTA (ORT, ROS, QUI) and NV (LIM, LRy, TBay) groups (POPGLAG = 1) previously reported (see Vera et al. (2019) for population codes). For this analysis, a burn‐in period of 100,000 steps followed by 200,000 Monte Carlo replicates was applied using the allele frequencies for reference populations from Vera et al. (2019). Fisher's exact test was performed to estimate the association between bonamiosis status (NV vs. LTA) of sampling locations and the percentage of individuals pertaining to the genetic clusters identified in the analyses (orange vs. blue; see Section 3).

Discriminant analysis of principal components (DAPC), a multivariant method to infer the number of clusters in a sample of genetically related individuals, was applied as a complementary method to infer structure in the samples studied using the Adegenet package function ‘dapc’ in RStudio (Jombart & Ahmed, 2011). A principal component analysis (PCA) from the matrix of genotypes was performed and then, a selected number of principal components (PCs) used as input for linear discriminant analysis (LDA). The selection of the optimal number of PCs to be further used in the LDA was done via cross‐validation, and those associated with the lowest root mean square error (RMSE) were retained. In addition, DAPCs retaining at least 90% of the cumulative variation of the data were evaluated.

### 2.5 Linkage disequilibrium

Pairwise linkage disequilibrium (LD) between outliers mapping in the same chromosome was estimated using the square of the correlation coefficient *r* ^2^ as implemented in the R package gaston v 1.5.7 with the ‘LD’ function (Perdry & Dandine‐Roulland, 2020). The results were represented in a LD heatmap plot using the same R package. A *p*‐value for estimated deviations from the null hypothesis (*r* ^2^ = 0) was obtained using an exact test for genotyping disequilibrium with Genepop 4.7 (Rousset, 2008). LD between consistent outliers was also estimated using the previous data set from Vera et al. (2019), considering that they represented the original reference and also the broader sampling area studied.

### 2.6 Mapping SNPs and differentially expressed genes in the flat oyster genome: functional enrichment assessment

A chromosome‐level flat oyster genome assembly generated and annotated at the Roslin Institute, University of Edinburgh (‘OEROSLIN’; NCBI Bioproject: PRJNA772111; Assembly accession: JAJSPN000000000; Gundappa et al., 2022) was used to map all SNPs and the 715 DEGs previously reported by Ronza et al. (2018). The genome assembly was 935.6 Mb in size, comprised of 1365 scaffolds (N50: 94.05 Mb), including 10 large scaffolds (sum: 875.78 Mbp; 93.6% of total genome assembly) corresponding to the haploid chromosome number of flat oyster (2*n* = 20; Leitao et al., 2002). All SNPs, including the consistent and suggestive outliers detected for resistance to bonamiosis, and the DEGs between NV and LTA oysters were mapped against the OEROSLIN genome to check for co‐localization. Functional enrichment of genes located at genomic regions associated with bonamiosis resistance was obtained with R package *GOfuncR* version 1.14.0 (Grote, 2021). Enriched Gene Ontology (GO) terms related to ‘Biological Process’, ‘Molecular Function’ and ‘Cellular Component’ and the associated genes were ascertained against the background of all genes in the flat oyster genome that received GO annotations.

## 3 RESULTS

The present study aimed to validate previous *Bonamia*‐resistance associated markers reported by Vera et al. (2019) in an independent narrower scenario following a population genomics approach and exploiting a new flat oyster genome assembly for a more comprehensive interpretation of results. Different SNP panels were used according to the goal of the analyses performed (Table 2). The whole SNP data set, including polymorphic and monomorphic markers, was used to estimate genetic diversity, which made possible comparisons with previous studies in flat oyster, while the neutral and outlier data sets were used to investigate genetic structure with respect to the differential bonamiosis pressure operating on flat oyster samples (i.e. NV vs. LTA). The number of polymorphic markers available for the different classes (i.e. neutral and outlier) was lower than reported by Vera et al. (2019) because of the smaller geographical range analysed. In particular, the number of suggestive and consistent SNP outliers dropped from 87 to 71 and from 21 to 16, respectively (Table 2).

**TABLE 2:** SNP panels used for the different analyses performed in *Ostrea edulis* from the North Sea

SNPs	SNP panels used	Genetic diversity	Population differentiation/structure
11,641 SNPs	Initial panel	✓	
10,591 SNPs	Neutral panel^a^		✓
71 and 16 SNPs	Outlier panel^b^	✓	✓

^a^ Only polymorphic loci.

^b^ Suggestive and consistent outliers according to Vera et al. (2019).

### 3.1 Genetic diversity

Observed heterozygosity (*H*_o_), expected heterozygosity (*H*_e_) and allelic richness (Ar) were estimated as averages across the 11,641 SNP data set obtained in the North Sea samples after filtering, considering their origin. In wild samples, *H*_o_ ranged between 0.264 in NO and 0.275 in OS (mean = 0.269 ± 0.003); *H*_e_ from 0.265 in NO to 0.271 in OS (0.268 ± 0.002); and Ar from 1.893 in WZH to 1.908 in OS and GBR (1.903 ± 0.005) (Table 3A). Hatchery samples showed significantly lower genetic diversity figures for all estimators than the wild ones (Ar = 1.903 vs. 1.833; *H*_e_ = 0.268 vs. 0.257; Kruskal‐Wallis tests; *p* = 0). All sampling locations showed no deviation from panmixia using the whole SNP data set (HWE; *p* > 0.05), although the intrapopulation fixation index (*F*_IS_) was negative and significant using the 95% range test in the hatchery samples, indicating a slight heterozygote excess. Nearly identical figures were obtained using neutral loci (data not shown).

**TABLE 3:** Genetic diversity of *Ostrea edulis* from the North Sea with the (A) whole SNP data set; (B) 16 consistent outliers; (C) 71 suggestive outliers

Sample	Bonam. status	Polym. SNPs	Ar	*H*_o_	*H*_e_	*F*_IS_	Lower/upper BC 95% CI
(A)							
OS	LTA	10,371	1.908	0.275	0.271	−0.013	−0.044 to 0.011
OSH	LTA	9471	1.839	0.271	0.259	−0.048	−0.080 to −0.025
GBR	LTA	9905	1.908	0.267	0.268	0.003	−0.020 to 0.020
WZH	NV	9050	1.827	0.273	0.254	−0.076	−0.115 to −0.047
NO	NV	10,132	1.893	0.264	0.265	0.002	−0.015 to 0.015

Sample	Bonam. status	Ar	*H*_o_	*H*_e_	*F*_IS_	Lower/upper BC 95% CI
(B)						
OS	LTA	2.000	0.266	0.273	0.024	−0.154 to 0.226
OSH	LTA	1.934	0.205	0.252	0.090	−0.069 to 0.267
GBR	LTA	2.000	0.206	0.252	0.184	−0.066 to 0.527
WZH	NV	1.435	0.110	0.114	0.039	−0.125 to 0.215
NO	NV	1.880	0.169	0.158	−0.076	−0.167 to 0.004

Sample	Bonam. status	Ar	*H*_o_	*H*_e_	*F*_IS_	Lower/upper BC 95% CI
(C)						
OS	LTA	1.972	0.301	0.301	−0.001	−0.099 to 0.109
OSH	LTA	1.903	0.265	0.267	0.006	−0.068 to 0.089
GBR	LTA	1.986	0.283	0.300	0.056	−0.047 to 0.174
WZH	NV	1.700	0.250	0.224	−0.115	−0.180 to −0.056
NO	NV	1.906	0.256	0.263	0.027	−0.032 to 0.080

*Note*: Sample codes are shown on Table 1.

The program Colony estimated a total of 16 breeders, six males and 10 females, and an effective population size (*N*_e_) of 11 for OSH, and 16 breeders, seven males and nine females, and *N*_e_ = 10 for WZS, suggesting a rather balance contribution of breeders. In fact, the average number of individuals per family was 1.474 ± 0.208 (19 families; range: 1–4) for OSH and 2.077 ± 0.380 (13 families; range: 1–7) for WZS. Conversely, wild samples showed no parentage between all specimens analysed in GBR and NO (in both *N*_e_ = 2,147,483,647), and only two half‐sibs among the 28 were detected in OS (*N*_e_ = 1512), consistent with the higher genetic diversity observed in wild samples.

Interestingly, when analysing genetic diversity in the same samples with the outlier SNP data sets, the pattern changed, and naïve samples (NV: NO and WZH) showed lower genetic diversity than those from those long‐term affected by bonamiosis (LTA: GBR, OS and OSH), specially for the 16 outlier data set (Ar = 1.658 vs. 1.978, *p* = 0.004; *H*_e_ = 0.136 vs. 0.259; *p* = 0.004) (Table 3B,C).

### 3.2 Genetic structure and differentiation

Low, but significant, genetic differentiation was observed for most pairwise comparisons between the five North Sea sampling locations with the neutral SNP data set (average pairwise *F*_ST_ = 0.0283; Table 4A). Among the wild locations, OS and GBR from the Netherlands were the most similar (*F*_ST_ = 0.0003), while the Norwegian (NO) samples were the most divergent (average *F*_ST_ = 0.0169). The two hatchery samples showed the highest pairwise *F*_ST_ value considering the neutral SNP data set (0.0540), but also a high average pairwise *F*_ST_ with respect to the wild ones (0.0356). Lower but not significant (*p* > 0.05) average pairwise *F*_ST_ values between all sampling locations were observed with the consistent (*F*_ST_ = 0.0253) and suggestive (*F*_ST_ = 0.0159) outliers (Table 4B,C) regarding the neutral data set (*F*_ST_ = 0.0283). However, they were higher than the neutral markers when comparing NV (NO and WZH) vs. LTA (GBR, OS and OSH) sampling locations (average pairwise *F*_ST_ = 0.0348 and 0.0289 for consistent and suggestive outliers, respectively), although not significant (*p* > 0.05).

**TABLE 4:** Pairwise *F*_ST_ values between populations of *Ostrea edulis* from the North Sea with SNP data sets: (A) 10,591 neutral; (B) 16 consistent outliers; (C) 71 suggestive outliers

	Bonam. status	OS	OSH	GBR	WZH	NO
(A)						
OS	LTA	–	0.0000	0.1848	0.0000	0.0000
OSH	LTA	**0.0228**	–	0.0000	0.0000	0.0000
GBR	LTA	0.0003	**0.0269**	–	0.0000	0.0000
WZH	NV	**0.0282**	**0.0540**	**0.0299**	–	0.0000
NO	NV	**0.0165**	**0.0409**	**0.0172**	**0.0463**	–

Sample	Bonam. status	Polym. SNPs	Ar	*H*_o_	*H*_e_	*F*_IS_	Lower/upper BC 95% CI
(B)							
OS	LTA	10,371	1.908	0.275	0.271	−0.013	−0. 044 to 0.011
OSH	LTA	9471	1.839	0.271	0.259	−0.048	−0.080 to −0.025
GBR	LTA	9905	1.908	0.267	0.268	0.003	−0.020 to 0.020
WZH	NV	9050	1.827	0.273	0.254	−0.076	−0.115 – −0.047
NO	NV	10,132	1.893	0.264	0.265	0.002	−0.015 to 0.015

	Bonam. status	OS	OSH	GBR	WZH	NO
(C)						
OS	LTA	–	0.9992	1.0000	0.0001	0.0000
OSH	LTA	−0.0111	–	0.9963	0.0000	0.0515
GBR	LTA	−0.0182	−0.0138	–	0.0001	0.2311
WZH	NV	**0.0491**	**0.0439**	**0.0401**	–	0.0002
NO	NV	**0.0219**	0.0112	0.0073	**0.0286**	–

*Note*: Pairwise *F*_ST_ values and their *p*‐values below and above the diagonal, respectively. Bold type indicates significant *F*_ST_.

**FIGURE 2:**
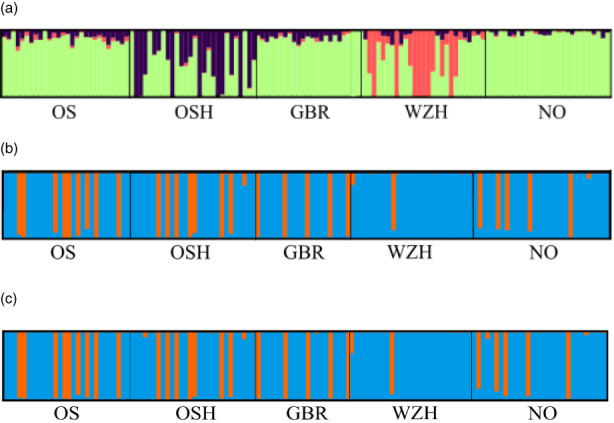
STRUCTURE analysis in *Ostrea edulis* from the North Sea with: (a) neutral for *K* = 3 and (b) 16 outlier, and (c) 71 outlier SNP data sets for *K* = 2. Each vertical bar represents a single individual and its colour proportion the posterior probability of its assignment to the defined STRUCTURE identified

The STRUCTURE analysis without a priori information assessed for *K* = 1–6 using deltaK and Mean LnP(K) estimators, rendered an optimal *K* = 3 with the neutral and *K* = 2 using both outlier SNP data sets. A clear distinction between the hatchery (WZH, OSH) and wild samples (NO, GBR, OS) was observed with the neutral data set (Figure 2a), while the wild samples constituted a single cluster for all Ks analysed. However, two consistent clusters (‘orange’ and ‘blue’) were observed in all sampling locations when the consistent and suggestive outlier data sets related to bonamiosis resistance were used (Figure 2b,c), the same ‘orange’ individuals being identified with both outlier data sets. Individuals pertaining to ‘orange’ and ‘blue’ clusters (occasionally with an admixed composition) were detected in all sampling locations, although at different frequencies, in accordance with the bonamiosis status of the sampling area (NV vs. LTA). WZH (NV) showed the lowest ‘orange’ proportion (3.7%), while OS (LTA) the highest (32.1%). A marginally significant association was detected between bonamiosis status (LTA: OS, GBR, OSH (77 individuals) vs. NV: NO, WZH (57 individuals)) and the percentage of ‘orange’ individuals (21 vs. 6; Fisher's exact test; *p* = 0.054).

**FIGURE 3:**
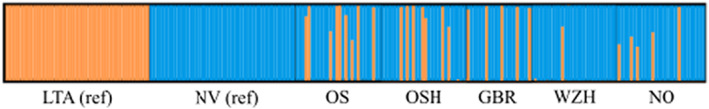
STRUCTURE analysis in *Ostrea edulis* from the North Sea (*N* = 134) for the 16 consistent outliers considering a priori information of long‐term affected (LTA, orange, *N* = 47) and naïve (NV, blue, *N* = 48) samples from Vera et al. (2019). Each vertical bar represents one individual, and the colour proportion for each bar represents the posterior probability of assignment of each individual to the defined STRUCTURE groups

To ascertain the correspondence between the ‘orange’ and ‘blue’ clusters with the NV and LTA groups previously reported by Vera et al. (2019), another STRUCTURE analysis with a priori information was performed with the consistent and suggestive outlier SNP data sets, respectively. Allelic frequencies of a pool of the NV samples (Figure 3, blue colour) and a pool of the LTA samples (orange colour) previously reported by Vera et al. (2019) were used as reference populations for the analysis. Results showed that all the 'orange’ and ‘blue’ individuals of the analyses without a priori information outlined before were fully associated with the LTA and NV references from Vera et al. (2019), respectively, both with the 16 (Figure 3) and 71 (not shown) outlier data sets, supporting its association with the bonamiosis status of samples.

**FIGURE 4:**
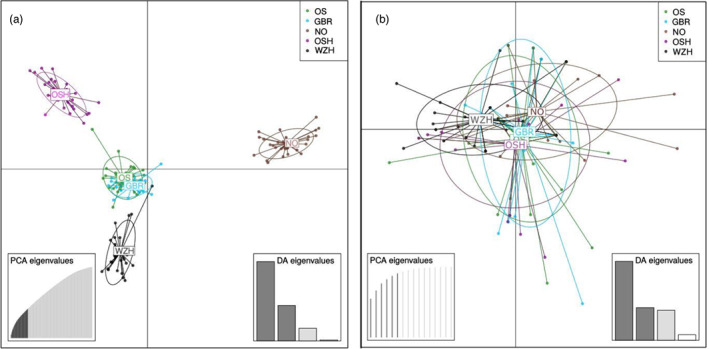
Discriminant analysis of principal components (DAPC) representation for *Ostrea edulis* from the North Sea (PCA1 abscissas, PCA2 ordinates). (a) Neutral panel with 101 PCs explaining 40.4% of the variance; (b) Consistent outlier data set (16 SNPs) with 6 PCs and 90.5% explained variance

The genetic structure of flat oyster samples was also tested with DAPC analyses (Figure 4). For the neutral panel, 101 PCs that explained 90.4% of the variance were used, and the higher differentiation component was found between sampling locations, with the wild NO being the most divergent (Figure 4a). For the consistent outlier panel (16 SNPs), we used 6 PCs that explained 90.5% of the variance (Figure 4b), and in this case, a great similarity was found between sampling locations, despite a much higher dispersion of individuals regarding the population centroids than observed with the neutral data set. In fact, the more distant individuals within each location mostly corresponded to the ‘orange’ samples detected in the STRUCTURE analysis, a pattern matching the barplot with *K* = 2. A very similar representation was observed with the suggestive outlier data set (data not shown).

From the STRUCTURE information, we split the whole sample into two subgroups pertaining to the two clusters observed with both outlier data sets: 27 ‘orange’ individuals associated to the LTA cluster and 107 ‘blue’ individuals to the NV one. For this, we applied the individual membership coefficient to each cluster (*q* value) using a threshold of *q*_“orange”_ > 0.5 and *q*_“blue”_ *≥* 0.5 to classify the few admixed individuals observed (Figure 2b,c). Genetic diversity (*H*_e_) was almost twofold higher in the ‘orange’ than in the ‘blue’ cluster with both outlier data sets, while the *F*_IS_ was negative and highly significant in the ‘orange’ cluster, indicating a heterozygote excess, but not in the ‘blue’ one, which conformed to Hardy–Weinberg expectations (Table 5). The deviation was nearly double for the consistent outlier data set (−0.376) than for the suggestive one (−0.196), while the ‘blue’ group did not depart from *F*_IS_ = 0 in both cases. Furthermore, we also performed a DAPC analysis with the orange and blue groups, *K* = 1 being obtained when using the neutral panel, while *K* = 2 being the most likely explanation when using both outlier data sets.

**TABLE 5:** Genetic diversity for the “orange” and “blue” groups identified in *Ostrea edulis* from the North Sea with STRUCTURE for the 16 (A) and 71 (B) outlier panels

Population	Ar	*H*_o_	*H*_e_	*F*_IS_	Lower and upper BC 95% CI
(A)					
Orange	2.00	0.590	0.430	−0.376	−0.503 to −0.234
Blue	1.91	0.270	0.270	−0.064	−0.149 to 0.028
(B)					
Orange	1.97	0.430	0.360	−0.196	−0.273 to −0.119
Blue	1.72	0.230	0.230	−0.009	−0.044 to 0.023

Furthermore, *F*_ST_ between ‘orange’ and ‘blue’ groups was 0.5118 for the consistent and 0.2274 for the suggestive outlier data sets (*p* < 0.001), while nonsignificant for the neutral (*F*_ST_ = 0.008; *p* > 0.05). The comparison of the *F*_ST_ distribution between the ‘orange’ and ‘blue’ samples per locus with the three data sets revealed sharp differences between the neutral and outlier data sets, especially for the consistent outliers (Figure S1). All neutral loci lied within the first interval (0–0.1), while the outlier loci distributed across the whole range up to 0.9, the highest modal proportions being between 0.7 and 0.8 for the consistent outliers. A similar frequency histogram per locus was constructed for the same data sets considering *F*_IS_ and again, a sharp difference was observed between the three data sets, especially for the outliers vs. neutral data sets (Figure S2). Neutral loci were normally distributed around *F*_IS_ = 0, while *F*_IS_ from outlier data sets was strongly biased towards negative values (71 outliers mean: −0.110; range: −0.919 to 0.784), especially for the consistent one (mean: −0.288; range: −0.919 to 0.076).

### 3.3 Genomic mapping of DEGs and outliers: linkage disequilibrium

**FIGURE 5:**
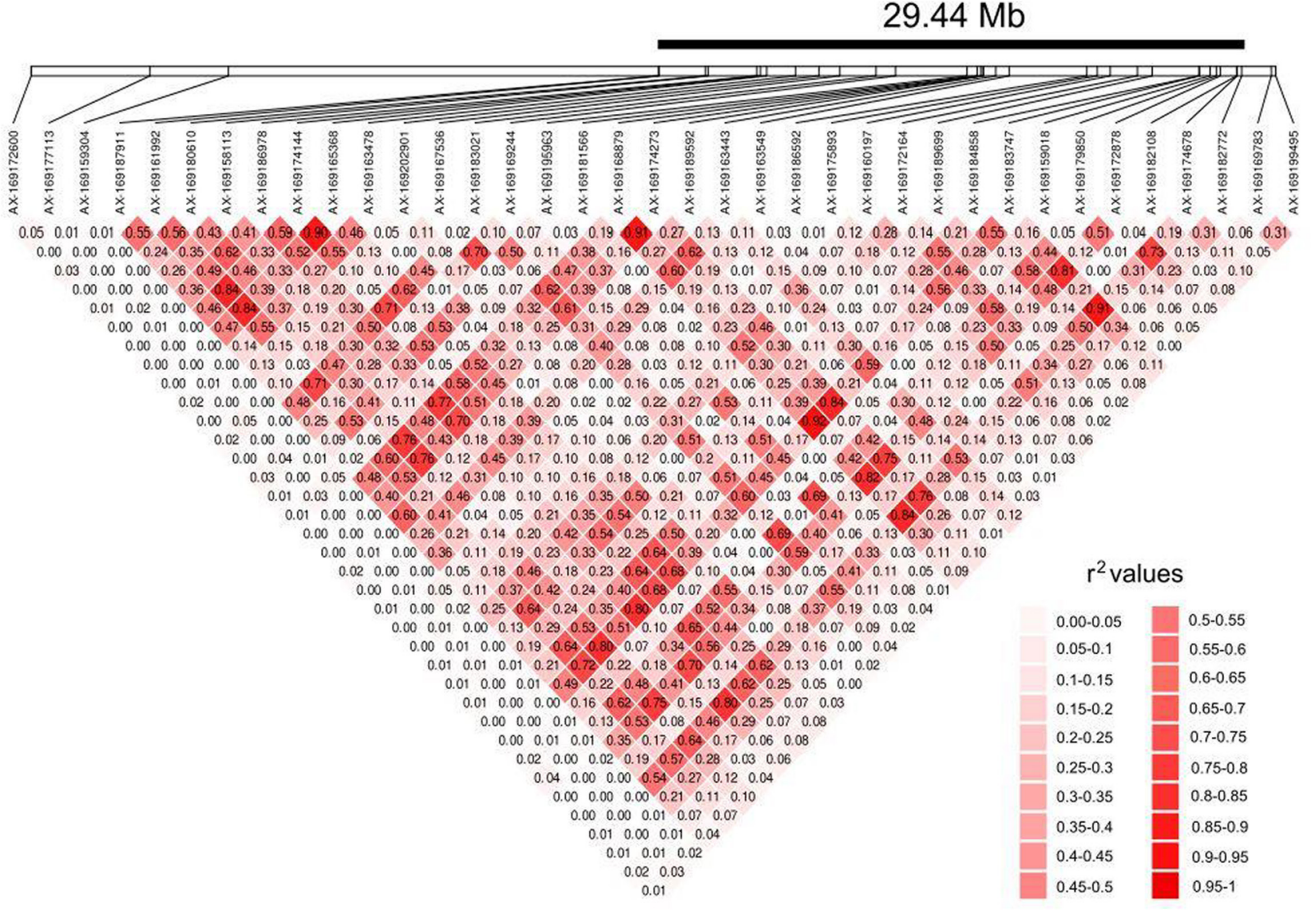
Linkage disequilibrium (*r* ^2^) between all pairs of suggestive outliers at OE‐C8 of Ostrea edulis. LD intensity (*r* ^2^ from 0 to 1) is shown using a range from whitish to reddish colours, respectively, as shown in the scale of the figure

**FIGURE 6:**
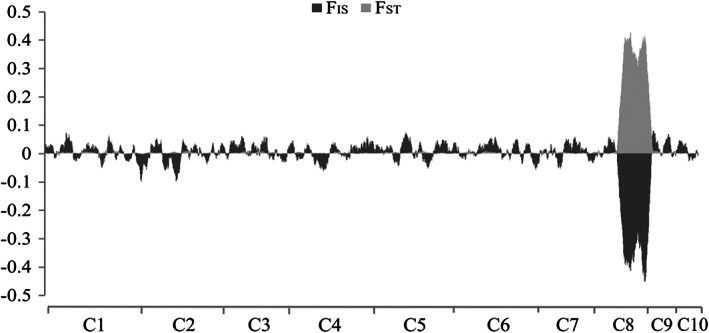
*F*_ST_ values between ‘orange’ vs. ‘blue”’groups and *F*_IS_ values within groups with all the 9277 SNPs mapping in the 10 *Ostrea edulis* chromosomes using average values of sliding windows of 50 SNPs across all chromosomes (C)

Using the flat oyster genome, we mapped the outliers associated with bonamiosis resistance along with the differentially expressed genes (DEGs) detected by Ronza et al. (2018) when comparing the response of NV and LTA strains to a bonamiosis challenge for an integrative analysis (Table S1A). An important proportion of the 71 suggestive and nearly all the 16 consistent outliers associated with the bonamiosis status of sampling locations mapped at OE‐C8 (37 and 14, respectively) (Table 6). We investigated LD between those 37 SNPs at OE‐C8, since a strong LD between outliers had been previously reported by Vera et al. (2019). Most outlier SNPs lied within a region encompassing 29.44 Mb (from AX‐169174678 to AX‐169187911), including the 14 most consistent, and showed LD between many markers across that region. Excluding AX‐189186592, which showed the lowest *F*_ST_ between ‘orange’ and ‘blue’ groups (0.182), all consistent outliers showed highly significant LD after Bonferroni correction (*r* ^2^ average: 0.554; range: 0.20–0.92; *p* < 0.00064; Figure 5). A very similar result was obtained excluding the same marker, when assessing LD for the consistent outliers at OE‐C8 using the broader sampling scenario previously reported by Vera et al. (2019) (*r* ^2^ range: 0.27–0.94; *p* < 0.00064; Figure S3). Interestingly, most SNPs located in that region showed highly significant *F*_ST_ values between ‘orange’ vs. “blue” groups and highly significant negative *F*_IS_ values within groups as compared to the whole genome background using average *F*_ST_ and *F*_IS_ values across sliding windows of 50 SNPs (Figure 6). This enabled us to refine the differential region between both groups up to 33.83 Mb (Table S1A) and suggested that several other SNPs in that region should likely be outliers between LTA and NV groups (Figure 6).

**TABLE 6:** Distribution of differently expressed genes (DEG) from Ronza et al. (2018) and outlier loci from Vera et al. (2019) per chromosome in the Ostrea edulis genome

Chromosome	Length (pb)	Total	DEG	DEG/total genes (%)	71 outliers	16 outliers
1	117,440,623	4750	84	1.768	10	–
2	101,867,661	3960	80	2.020	4	1
3	101,833,125	3720	82	2.204	2	–
4	99,930,069	4022	90	2.238	7	–
5	95,564,955	3720	80	2.151	1	–
6	94,056,450	3743	73	1.950	3 (1)	–
7	84,932,467	3167	68	2.147	1	1
8	70,328,625	2714	65	2.395	37	14
9	65,180,066	2512	29	1.154	1 (1)	–
10	44,655,554	1520	30	1.974	2	–
No match			30		3	–

*Note*: In parentheses two outliers with the same matching score in two different chromosomes counted twice.

Moreover, among the 711 differentially DEG reported by Ronza et al. (2018) mapping on the flat oyster genome, 65 were located on OE‐C8, representing the highest gene density (DEGs/total genes; Table 6), although nonsignificant when tested across all flat oyster chromosomes (chi‐square; *p* = 0.170). Among them, a block of 1.4 Mb (from 26.3 to 27.7 Mb), close to the proximal end of the 29.44 Mb region, included several genes involved in immune or stress responses, namely genes encoding three collagen‐alpha proteins, calpain, programmed cell death protein 10, mt rRNA methyltransferase 2 and G‐coupled protein coupled receptor Mth2 (Table S1A). Three additional methyltransferase genes were detected within the 29.44 Mb region along with genes with functions linked to endo‐ and exocytosis and cell adhesion.

We also investigated the overrepresented GO terms on genes from OE‐C8 in comparison to the flat oyster genome background. Enriched biological processes included those with immune functions, such as interleukin‐4‐mediated signalling pathway, negative regulation of natural killer cell activation, regulation of peptidoglycan recognition protein signalling pathway, negative regulation of response to interferon‐gamma and chromosome segregation (Table S2). Terms associated with interleukin‐4 signalling and response to interferon‐gamma were explained by genes encoding histone‐lysine N‐methyltransferase setd1b and multiple copies of Poly (ADP‐ribose) polymerase member 14. Several genes encoding peptidoglycan recognition proteins 1 and 2, along with lectin galactoside‐binding soluble protein 4 were responsible for the enrichment of terms associated with natural killer cell activation and peptidoglycan recognition signalling. Finally, the enriched term ‘chromosome segregation’ was explained by a large set of genes, including those encoding anaphase‐promoting complex 23, structural maintenance of chromosome protein 4, ATP‐dependent DNA helicase MER3, cyclin‐B3, meiosis sister chromatid cohesion complex protein, mitotic spindle checkpoint protein Bub3, MMS19 excision repair mechanism protein, dynactin subunit 1 and retinoblastoma‐associated protein.

## 4 DISCUSSION

European flat oyster production is severely threatened in the Northeast Atlantic by the intracellular protozoan *B*. *ostreae*, (Engelsma et al., 2014). Within a very low, but significant genetic differentiation scenario in the Atlantic area (*F*_ST_ = 0.0079; Vera et al., 2016), flat oyster populations that were never in contact with the parasite (naïve: NV) coexist with populations affected for many generations (long‐term affected: LTA), dating back to the first report of the parasite in Europe (Comps et al., 1980; Pichot et al., 1980). This observation suggests that while larvae can be infected by the parasite (Arzul et al., 2011), its transmission to other areas is not ensured. Vera et al. (2019) took advantage of the patchy distribution of the parasite in the Atlantic area to identify 21 consistent and 87 suggestive outliers putatively associated with divergent selection to bonamiosis by analysing genetic differentiation between three NV and three LTA flat oyster beds distributed across the three different genetic units reported in the species (Vera et al., 2016) using a SNP array including 14,950 putative SNPs (Gutiérrez et al., 2017). Furthermore, they reported strong linkage disequilibrium (LD) between many of those outliers, suggestive of a major QTL underlying resistance to the parasite. A deeper genomic analysis was essential to validate these observations that could be potentially applied to obtain *B*. *ostreae* resistant strains to recover flat oyster beds on European coasts. Consequently, the main goal of our study was to validate previous findings by Vera et al. (2019) in an independent sampling scenario and to integrate all previous functional and structural genomic information within the new assembled flat oyster genome. Also, we obtained the first family composition of hatchery batches for refining breeding protocols and devising the best strategy for maintaining genetic diversity in future restocking programs.

Average genetic diversity in wild samples from the North Sea (Ar = 1.875; *H*_e_ = 0.263) was very similar to that reported in the Northeast Atlantic using the same SNP data set by Vera et al. (2019). The lower number of polymorphic loci in our study is likely explained by the smaller area of collection and the low yet significant structure reported for this species in the Northeast Atlantic with microsatellites (*F*_ST_ = 0.0079; Vera et al., 2016) and SNPs (*F*_ST_ = 0.0061; Vera et al., 2019). As expected, since hatchery samples came from a single batch, average genetic diversity of hatchery batches was significantly lower (Ar = 1.833; *H*_e_ = 0.257) than their wild counterparts, congruent with the estimated effective population size (*N*_e_: between 10 and 11 for hatchery samples, and between 1512 and 2,147,483,647 for wild samples). Anyway, parental contributions were rather evenness both in OSH and WZH, which suggests that the reproduction protocol, at least regarding the maturation condition of parents, seems suitable, and so the main point to be considered in future restocking programs would be the number of batches and progenitors per batch to maintain diversity.

Population structure using neutral and outlier loci data sets was evaluated to disentangle genetic differentiation due to demographic factors (genetic drift and migration) from those related to selection due to bonamiosis pressure. As reported by Vera et al. (2019), low genetic differentiation was detected with neutral SNPs in the wild locations (average pairwise *F*_ST_ = 0.0113), although related to geographical distance, supporting isolation by distance. Divergence was much higher between hatchery and wild samples (average pairwise *F*_ST_ = 0.0325), as expected due to genetic drift associated to the low number of breeders used for batch production and STRUCTURE analysis depicted a clear differentiation between both groups.

Outlier data sets identified two genetic clusters (‘blue’ and ‘orange’) in the samples from the North Sea that were well defined at the individual level. The proportion of ‘orange’ and ‘blue’ individuals was marginally associated with their bonamiosis status (NV vs. LTA; *p* = 0.054), the orange cluster being more frequent in the LTA samples. The sample size used in our study limits the statistical power of the Fisher's test and in fact, a highly significant association has been detected when sample size increased (LTA sample: 192 individuals (48 ‘orange’/144 ‘blue’); NV sample: 189 individuals (7 ‘orange’/182 ‘blue’); exact test *p*‐value <0.00001; Kamermans P., in preparation). Furthermore, the ‘orange’ group corresponded to the LTA group reported by Vera et al. (2019), suggesting its association with bonamiosis resistance. However, the clear‐cut structuring of individuals (‘orange’ or ‘blue’) in this study was not as evident in Vera et al. (2019), where many individuals with mixed composition were observed. It might be speculated that the broader geographical scenario assessed by Vera et al. (2019) is responsible for that observation and indeed, NV and LTA samples were separated in their DAPC analysis both with the consistent and suggestive outlier data sets, while in the North Sea samples appeared as a single cluster with many outliers related to ‘orange’ individuals.

‘Orange’ and ‘blue’ groups from the North Sea displayed striking genetic differentiation (*F*_ST_ = 0.5118 and 0.2266 for the consistent and suggestive outlier data sets, respectively) and the ‘orange’ group showed twice the genetic diversity of the ‘blue’, coupled with a highly significant heterozygote excess, while the ‘blue’ group did not depart from Hardy–Weinberg expectations. This observation might suggest an overdominance mechanism underlying resistance to bonamiosis, but further work should be done for its confirmation. Tolerance or resistance to stress or pathologies has been associated with overdominance in other studies (Di et al., 2015; Gallaga‐Maldonado et al., 2020; Maynard et al., 2016), sometimes involving specific immune genes, such as the histocompatibility complex (MHC; Kekäläinen et al., 2009; Penn et al., 2002). It is important to note that the term resistance applied until now in this and previous studies related to bonamiosis infection is rather inaccurate, as the response to the parasite may be related both to resistance against infection and the ability to survive once infected (tolerance) (Holbrook et al., 2021). A more appropriate term encompassing both components is resilience, and we suggest its use until more refined information is available. Preliminary data in the same area indicates that the orange individuals can be infected by *B*. *ostreae* (Kamermans P., unpublished data), but their survivorship could not be tested, and we do not know if they are actually more resistant to infection. To disentangle the basis for resilience of orange individuals, it would be necessary to devise a challenge at indoor facilities or alternatively, to gather broad field data with detailed quantitative information on parasite load along with genotyping of the associated genetic markers.

Vera et al. (2019) reported strong LD for outlier loci between NV and LTA beds and suggested a major QTL might underlie resilience to bonamiosis. By exploiting a new flat oyster genome, we demonstrated that nearly all consistent outliers are located on OE‐C8, spanning a region of 29.44 Mb and that half of the suggestive ones also mapped at OE‐C8, but population data suggest that this region could span 33.83 Mb, nearly half of OE‐C8. Previous studies suggested quick dropping of LD with physical distance in molluscs, especially in the wild, associated with their typical large effective population sizes (Gutiérrez et al., 2017; Hu et al., 2021; Vera et al., 2022; Zhong et al., 2017). In edible cockle (*Cerastoderma edule*), a species with an overlapping distribution and similar life‐history features to flat oyster, Vera et al. (2022) detected a maximum *r* ^2^ of 0.05, which was significant at distances below 50 kb. The LD detected at OE‐C8 in flat oyster suggests a chromosome reorganization blocking recombination, such as an inversion, that could be confirmed by comparing whole genome long‐read re‐sequencing of ‘orange’ and ‘blue’ samples. Polymorphisms for inversions have been reported to be maintained by natural selection as adaptations to specific environmental conditions by establishing blocks of coadapted genes in a diverse range of species (Fontdevila et al., 1983; Maroso et al., 2018; Mérot et al., 2021).

Flat oyster responses to *B*. *ostreae* have been associated with preventing the entrance of the parasite into haemocytes, and with apoptotic processes arresting proliferation of the parasite once it surpasses the external barrier (Gervais et al., 2016; Morga et al., 2012; Ronza et al., 2018). In our study, a set of 65 DEGs between naïve and long‐term affected strains (Ronza et al., 2018) were located on OE‐C8, representing the highest proportion among the 10 flat oyster chromosomes. To note, a block constituted by five genes close to the 33.83 Mb refined region at OE‐C8 included several collagen‐related genes involved in the extracellular matrix (external barrier of haemocytes) and a programmed cell death protein (avoidance of parasite proliferation). Interestingly, a SNP associated to the collagen‐IV DEG (Ronza et al., 2018) was the only outlier associated with divergent selection among the 37 candidate genes evaluated by Vera et al. (2019). Also, several methyltransferases involved in epigenetic patterns were detected at OE‐C8 and could explain the faster response of LTA oysters to bonamiosis reported by Ronza et al. (2018), considering the putative transmission of epigenetic patterns across generations (Eirín‐López & Putnam, 2019). Furthermore, the functional enrichment analysis performed on OE‐C8 also identified GO terms such as interleukin‐4 regulation signalling pathway involving five genes related to epigenetic marking and a set of 26 genes related to chromosome segregation, many of them associated with apoptotic defence mechanisms.

## 5 CONCLUSION

Our data adds new evidence to the divergent selection outliers detected previously by Vera et al. (2019) associated with the bonamiosis status of samples on an independent sample collection. Also, the suggested genomic region associated with bonamiosis resistance by the same authors was supported and most outliers were located on a region of OE‐C8. These outliers displayed a strong LD over a large chromosomal region, which supports a chromosome reorganization at OE‐C8 that could be maintained by overdominance related to *B. ostreae* resilience. This genomic region include genes encoding proteins related to apoptosis and extracellular matrix components, considered key in the response to an intracellular infection. We also detected significant enrichment GO terms involving genes controlling epigenetic marks that could be related to the quick response to bonamiosis observed in LTA oysters. The identified markers at OE‐C8 associated with *B*. *ostreae* resilience could aid for recovering its production and ecosystem services throughout the European coast. However, further work is needed to test this association at individual level, to disentangle the components underlying bonamiosis resilience and to confirm the chromosome reorganization suggested at OE‐C8 supporting an overdominance mechanism. Finally, the putative involvement of epigenetic mechanisms on the transgenerational immune memory for a quick response to the parasite is a suggestive hypothesis that deserves further attention.

## CONFLICT OF INTEREST

The authors declare that they have no competing interest.

## Supporting information


Figures S1–S3

Table S1

Table S2

## Data Availability

All genotyping information of this manuscript including neutral, outlier and bonamiosis‐associated markers is available at the Dryad Repository https://doi.org/10.5061/dryad.s1rn8pkbp. [Correction added on 30 July 2022, after first online publication: The Data Availability Statement has been corrected in this version.]
