# Supplementary material for: A single genomic region involving a putative chromosome rearrangement in flat oyster (Ostrea edulis) is associated with differential host resilience to the parasite Bonamia ostreae
Source: Evol Appl. 2022 Jul 21;15(9):1408–22. doi: 10.1111/eva.13446 (PMC9488685; doi:10.1111/eva.13446)
Supplement: Supplementary file 1 — Figures S1–S3 [file EVA-15-1408-s002.docx]

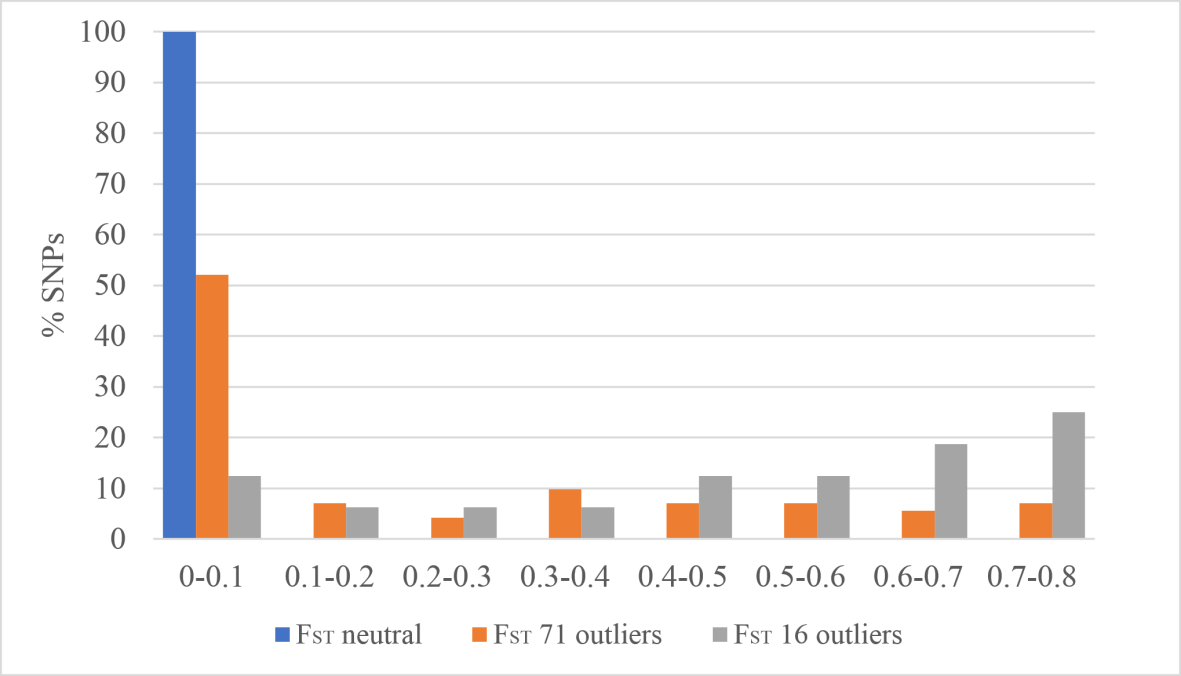


**Supplementary Figure 1.** Frequency histogram showing the number of outliers per F_ST_ interval using the neutral, the 71 suggestive and 16 consistent SNP outlier panels between NV and LTA samples of *Ostrea edulis* from the North Sea.


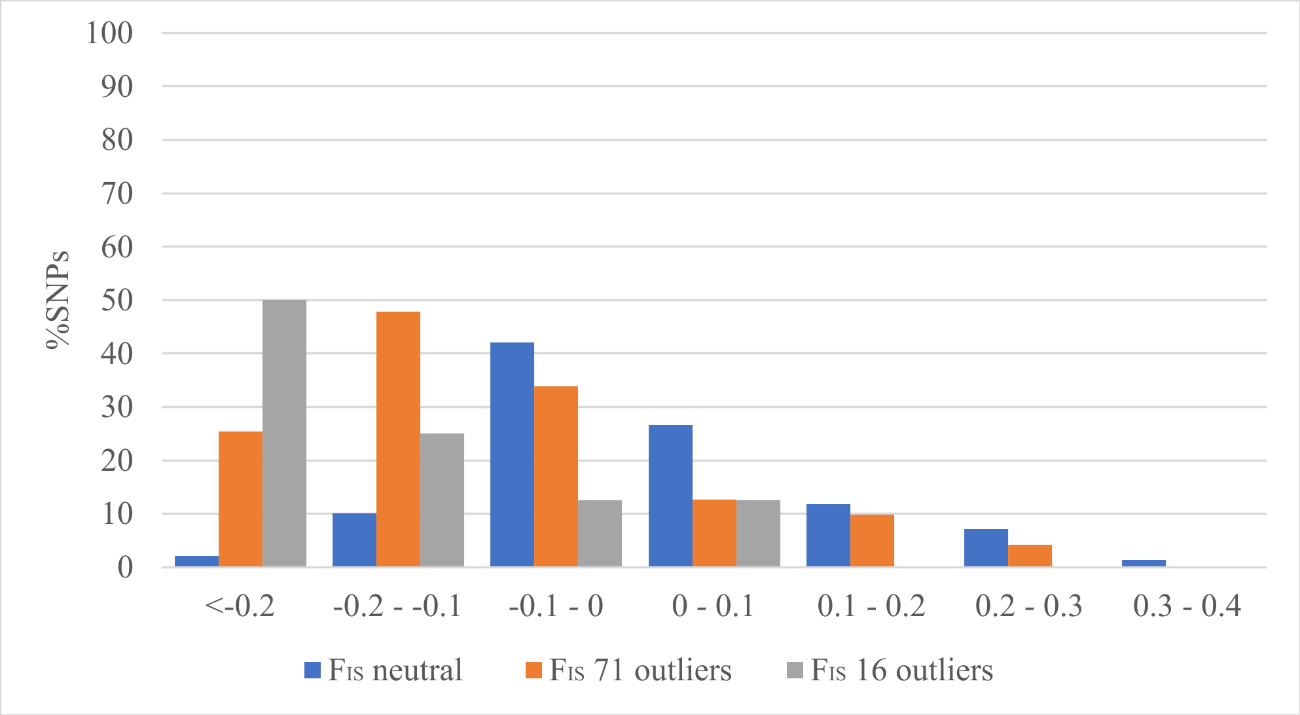


**Supplementary Figure 2.** Comparative frequency histogram of the number of outliers per F_IS_ interval using the neutral, the 71 suggestive and 16 consistent SNP outlier panels within NV and LTA samples of *Ostrea edulis* from the North Sea


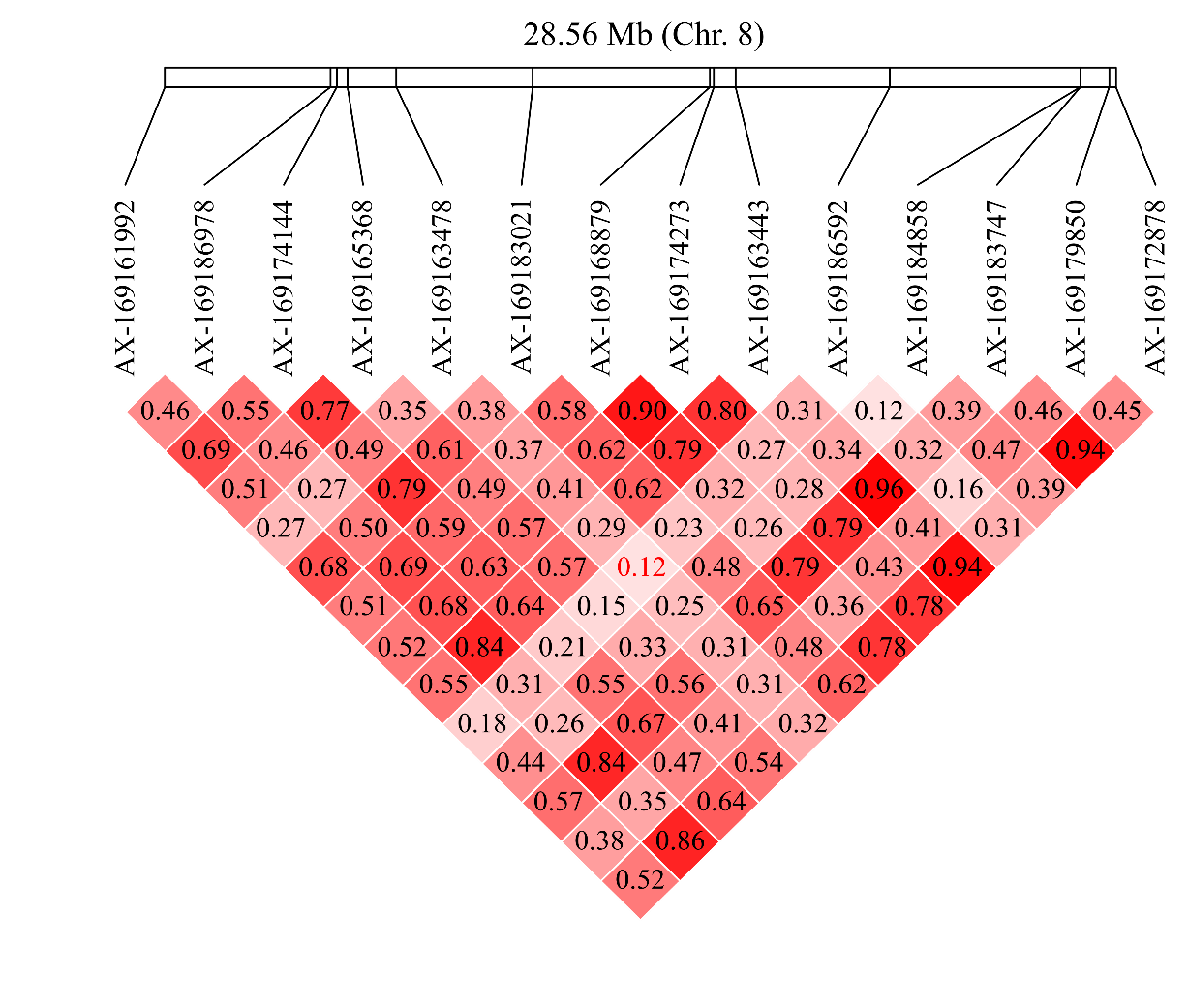


**Supplementary Figure 3**. Linkage disequilibrium (r^2^) between all pairs of consistent outliers at OE-C8 of Ostrea edulis. LD intensity (r^2^from 0 to 1) is shown using a range from whitish to reddish colours, respectively. All r^2^ values significant after Bonferroni correction (P < 0.00005), except that highlighted in red font.
